# Motor–Language Coupling in Huntington’s Disease Families

**DOI:** 10.3389/fnagi.2014.00122

**Published:** 2014-06-12

**Authors:** Lucila Kargieman, Eduar Herrera, Sandra Baez, Adolfo M. García, Martin Dottori, Carlos Gelormini, Facundo Manes, Oscar Gershanik, Agustín Ibáñez

**Affiliations:** ^1^Laboratory of Experimental Psychology and Neuroscience (LPEN), Institute of Cognitive Neurology (INECO), Favaloro University, Buenos Aires, Argentina; ^2^National Scientific and Technical Research Council (CONICET), Buenos Aires, Argentina; ^3^UDP-INECO Foundation Core on Neuroscience (UIFCoN), Diego Portales University, Santiago, Chile; ^4^Universidad Autónoma del Caribe, Barranquilla, Colombia; ^5^School of Languages, National University of Córdoba (UNC), Córdoba, Argentina; ^6^Australian Research Council (ARC) Centre of Excellence in Cognition and its Disorders, Sydney, NSW, Australia

**Keywords:** Huntington’s disease, action–language, ACE, KDT, motor–language coupling, familial vulnerability

## Abstract

Traditionally, Huntington’s disease (HD) has been known as a movement disorder, characterized by motor, psychiatric, and cognitive impairments. Recent studies have shown that motor and action–language processes are neurally associated. The cognitive mechanisms underlying this interaction have been investigated through the action compatibility effect (ACE) paradigm, which induces a contextual coupling of ongoing motor actions and verbal processing. The present study is the first to use the ACE paradigm to evaluate action–word processing in HD patients (HDP) and their families. Specifically, we tested three groups: HDP, healthy first-degree relatives (HDR), and non-relative healthy controls. The results showed that ACE was abolished in HDP as well as HDR, but not in controls. Furthermore, we found that the processing deficits were primarily linguistic, given that they did not correlate executive function measurements. Our overall results underscore the role of cortico-basal ganglia circuits in action–word processing and indicate that the ACE task is a sensitive and robust early biomarker of HD and familial vulnerability.

## Introduction

Huntington’s disease (HD) is an autosomal dominant neurodegenerative disorder resulting from the expansion of CAG trinucleotide repeats (36 or more repeats) within a gene on the short arm of chromosome 4, which codes for the protein huntingtin (Ho et al., 2001). HD is clinically diagnosed by symptoms such as chorea, bradykinesia, dystonia, and incoordination (Tröster, 2006). These typically become evident between the ages of 35 and 44, but, depending on the number of CAG repeats, disease onset ranges from childhood to late adulthood (Bates and Jones, 2002). Due to developments in genetics, HD can now be diagnosed in the absence of motor symptoms. However, in pre-HD and early HD stages, the pathology may be present without any of its typical (motor, psychiatric, or otherwise cognitive) signs (Stout et al., 2011), which underscores the need for more sensitive measures. In such cases, damage is usually restricted to basal ganglia structures, especially the caudate nucleus (Harris et al., 1999; Kipps et al., 2005; Kloppel et al., 2008; Henley et al., 2009). As in other neurodegenerative disorders (Ibanez and Manes, 2012), early detection would enable more effective diagnosis and treatment.

Recent reports of other motor disorders in their early-stages have found deficits in verbal domains, including action–language. This is true of progressive supranuclear palsy (Bak et al., 2005), frontotemporal dementia (Rhee et al., 2001; D’Honincthun and Pillon, 2008), Parkinson’s disease, and amyotrophic lateral sclerosis (Neary et al., 2000; Bak et al., 2001; Peran et al., 2003, 2009; Bak and Hodges, 2004; Boulenger et al., 2008). Thus, action–language deficits are an emergent agenda in motor conditions.

In HD, typical motor alterations and cognitive deficits (Bachoud-Levi et al., 2001; Montoya et al., 2006) are accompanied by disorders in verbal production (Podoll et al., 1988; Murray and Lenz, 2001), including reduced verbal fluency (Chenery et al., 2002; Ho et al., 2002; Azambuja et al., 2012), affixation errors (Ullman et al., 1997), and syntactic processing difficulties (Teichmann et al., 2005, 2008). Language comprehension deficits have also been reported (Teichmann et al., 2008; Azambuja et al., 2012). Moreover, late-stage HD has been associated with action–verb generation deficits (Peran et al., 2004), and action naming was found to be the task that best discriminated controls and HD patients (HDP) (Azambuja et al., 2012). Nevertheless, the relationship between action–language and motor action remains poorly explored.

Nowadays, research into action–verb processing has focused on frontal and motor cortices (Federmeier et al., 2000; Pulvermuller et al., 2001; Yokoyama et al., 2006; Boulenger et al., 2008; Cappelletti et al., 2008; Kemmerer et al., 2008; Tomasino et al., 2008). However, the basal ganglia may also be involved in both motor representation and initiation of semantic integration during action–verb processing (Cardona et al., 2013). In this sense, a crucial unresolved issue is whether subcortical motor networks are actively engaged in action–language. The present study addresses this question by examining motor–language coupling during sentence comprehension in HDP. Furthermore, we explore this sentence comprehension mechanism in asymptomatic HD patients’ relatives (HDR). Cognitive impairments have been documented in HDP during pre-clinical stages near the onset of disease (Lawrence et al., 1998a). Moreover, biological, motor, and cognitive vulnerability have also been reported in HDR (Markianos et al., 2008; Dorsey, 2012). Building on these findings, we evaluated (a) whether motor–language coupling is affected by subcortical motor affectation, and (b) whether this coupling is a marker of early-stage HD or HD vulnerability. To the best of our knowledge, this is the first study on motor–language coupling in HDP and their relatives.

## Materials and Methods

### Subjects

In total, 73 subjects received a full protocol evaluation (see Table 1 for demographic and clinical data).

**Table 1:** **Demographic and clinical data**.

	HDP	HDR	HDP vs. CTR	HDP– CTR	HDR– CTR
*N* (female/male)	18 (6/12)	19 (13/6)	NS	18 (6/12)	NS
				18 (12/6)	
Handedness (right/left)	17/18	19/19	NS	18/18	NS
				18/18	
Age (mean ± SD)	43 ± 10	29 ± 9	NS	43 ± 10	NS
				29 ± 9	
Level of education (mean ± SD)	9.5 ± 5	11.5 ± 2.7	NS	9.5 ± 5	NS
				11.5 ± 2.7	
Disease duration years (mean ± SD)	3.55 ± 3.014				
Onset disease years (mean ± SD)	39.72 ± 8.22				

*HD, HDR, and control groups*.

#### Huntington’s disease patients group

The HDP group consisted of 18 symptomatic patients genetically and clinically diagnosed with HD. They presented both clinical and cognitive manifestations as well as a positive family history of HD, according to the criteria proposed by Folstein et al. (1986). Patients with different neurological or psychiatric signs, or structural brain abnormalities compatible with diagnoses other than HD, were excluded from the study. To evaluate daily-life functional capacity in HDP, we used the Shoulson–Fahn Functional Capacity Scale (HDFCS) (Shoulson and Fahn, 1979). This instrument measures independence in daily activities such as dressing, eating, managing personal finances, and engagement in occupation. Functional capacity is ranked from 0 to 13 points, the latter score representing the most independent level of function (Shoulson and Fahn, 1979). Most of the participants in the HDP group were off medication (73%). Only five patients were under fluoxetine, clonazepam, and tetrabenazine treatment. Patients and relatives were recruited from the small rural town of Juan de Acosta, Colombia, a region possessing the second largest concentration of individuals with HD in the world (Pradilla et al., 2003).

#### Huntington’s disease relatives group

The HDR group was composed of 19 subjects with a positive family history of HD to the first-degree of consanguinity. They did not present any HD symptoms, and had not been diagnosed with HD or other neuropsychiatric diseases. Both the HDP and the HDR groups underwent a neurological examination and were assessed using the unified Huntington’s disease rating scale (UHDRS) (Huntington Study Group, 1996). UHDRS total motor scores were >5 for HDP and <5 for HDR, resembling the scores reported by Tabrizi et al. (2009). Patients and relatives had no history of any other major neurological illness, psychiatric disorders, or alcohol/drug abuse.

#### Control groups

Thirty-seven healthy comparison subjects were recruited and assigned to two control groups. One (*n* = 18) was matched with HDP and the other (*n* = 19) with HDR. Matching criteria were sex, age (±2 years), and years of education (±2 years). The HDP control group (HDP–CTR) had a mean age of 43.22 (±10.530), and a mean educational level of 10.16 (±4.218) years of schooling. For the HDR control group (HDR–CTR), the mean age was 29.50 (±10.245) and the mean level of education was 11.44 (±2.661) years of schooling. None of the subjects had a history of neurodegenerative disease, psychiatric disorders, or drug abuse. The demographic, clinical, and neuropsychological characteristics of the participants are summarized in Table 1. All participants gave written informed consent in agreement with the Helsinki declaration. Also, the study was approved by the Ethics Committee of the Institute of Cognitive Neurology.

### Clinical and neuropsychological assessment

All participants completed a series of psychiatric questionnaires to establish a clinical symptom profile. The Beck depression inventory-II (BDI-II) (Beck et al., 1996) was used to rate depression. Anxiety symptoms were assessed by means of the Hamilton anxiety rating scale (HAM-A) (Hamilton, 1959). In addition, the Montreal cognitive assessment (MOCA) (Nasreddine et al., 2005) was used to assess the participants’ overall cognitive state, including short-term memory, visuospatial/executive skills (e.g., alternation, phonetic fluency, and abstraction), attention, working memory, language, and orientation.

Furthermore, all participants completed the INECO frontal screening (IFS) test (Torralva et al., 2009), which has been shown to successfully detect executive dysfunction (Torralva et al., 2009; Gleichgerrcht et al., 2011). The IFS includes the following eight subtests: (1) motor programing (Luria series, “fist, edge, palm”); (2) conflicting instructions (subjects are asked to hit the table once when the examiner hits it twice, or vice versa); (3) motor inhibitory control; (4) numerical working memory (backward digit span); (5) verbal working memory (months backwards); (6) spatial working memory (modified Corsi tapping test); (7) abstraction capacity (inferring the meaning of proverbs); and (8) verbal inhibitory control (modified Hayling test). The maximum possible score on the IFS is 30 points. Additionally, participants were evaluated with the Wechsler abbreviated scale of intelligence (WASI). This test includes vocabulary and similarities subtests and provides a verbal estimated IQ (Wechsler, 1999). Finally, participants were also administered the WAIS-III similarities subtest (Wechsler, 1997) to evaluate abstract thinking, and the Stroop test (Treisman and Fearnley, 1969) to assess mental speed, selective attention, and inhibitory control. To control for the influence of clinical symptoms (depression and anxiety) or cognitive state on experimental tasks, we applied ANCOVA tests adjusted for BDI-II, HAM-A, and MOCA scores. We report only those effects that were still significant after covariation.

### Experimental tasks

#### Semantic association of nouns

To assess the semantic association of nouns, we employed the picture version of the Pyramids and Palm Trees Test (PPT) (Howard et al., 1992). The PPT consists of 52 triplets of pictures depicting different objects. Each triplet is composed of a cue object–picture (e.g., spectacles) and two semantically related pictures (e.g., eye and ear). Participants are asked to point to the picture that is most closely related to the cue. Their goal is to discover the relation between the cue and the response picture, which varies across trials.

#### Action–verb processing

To assess selective action–verb processing, we used the picture version of the kissing and dancing test (KDT) (Bak and Hodges, 2003). The KDT uses 52 triads of images to assess access to semantic representations of verbs. It has proven useful in detecting subtle impairments in other subcortical motor diseases, such as Parkinson’s disease (Cardona et al., 2013; Ibanez et al., 2013).

#### Action sentence compatibility effect

We evaluated the interaction between language and motor processes using the action compatibility effect (ACE) paradigm. The ACE paradigm is well-suited for this study, since it recruits both motor and semantic brain areas, and has proved sensitive to deficits in other subcortical motor disorders (e.g., Parkinson’s disease) (Aravena et al., 2010; Ibanez et al., 2013). Moreover, the ACE task is unaffected by peripheral motor impairments (Cardona et al., 2014), which further underscores its relevance to the present study. Participants listened to sentences that described an action performed with the hand in a particular shape (open: OH, *n* = 52, or closed: CH, *n* = 52), as well as neutral sentences alluding to non-manual actions (neutral: N, *n* = 52). Participants indicated as quickly as possible when they understood each sentence by pressing a button with a pre-assigned hand-shape (open and closed; counterbalanced in two blocks). The combination of response type and sentence-type generates compatible (OH sentence and OH response or CH sentence and CH response), incompatible (OH sentence and CH response or vice versa), and neutral (N sentence with either response) trials. The ACE is defined as a longer reaction time (RT) in the incompatible than in the compatible conditions (Glenberg and Kaschak, 2002; Masumoto et al., 2004; Borreggine and Kaschak, 2006; Glenberg, 2006; Glenberg et al., 2008a,b; Aravena et al., 2010).

All participants performed the task with their dominant hand, although both hands were positioned in the required shape. By controlling the position of both hands, we controlled for possible bilateral manual interference, since posture modulates semantic processing (Glenberg et al., 2008b; Lindeman et al., 2008; van Elk et al., 2008; Badets et al., 2010). To ensure that all participants had understood the meaning of the sentences, they were asked to complete an offline questionnaire after finishing the task.

### Data analysis

For statistical analysis, we used the repeated measures analysis of variance (ANOVA) and χ^2^ for neuropsychological assessment. In the ACE paradigm, mean RTs were calculated for each subject for each type of trial (compatible, incompatible, and neutral) and each type of sentence (OH, CH, and N). Single trials eliciting outlier values with RTs outside 2.5 SD were excluded from the analyses. A mixed-repeated measure ANOVAs had group as a between-subject factor (HDP vs. controls, HDR vs. controls) and compatibility (compatible, incompatible, and neutral) as a within-subject factor. An additional factor was introduced, namely sentence-type (N, OH, and CH). Moreover, RTs in ACE were normalized by subtracting the mean RT of the neutral trials from the mean RTs of the compatible and incompatible trials. The N sentences are more predictable and frequent than OH and CH sentences, eliciting shorter RTs (Aravena et al., 2010; Ibanez et al., 2013). If either HDP or HDR evidences preserved sentence-type modulation (shorter RTs for N than OH and CH sentences), then the ACE in HD cannot be explained as a general motor impairment or as an artifact of response variability (Aravena et al., 2010; Ibanez et al., 2013; Cardona et al., 2014). Tukey’s HSD test was used in the calculation of *post hoc* contrasts.

We further explored individual differences in ACE; a global score of the ACE was defined by the subtraction of the mean RT for the incompatible and compatible conditions (Ibanez et al., 2013; Cardona et al., 2014). In the patient group, these global scores were tested for correlation with age and years of illness through Spearman’s rank correlations. We also performed Spearman’s rank correlations between the ACE and IFS total scores to evaluate involvement of executive functions in all groups.

## Results

### Demographic and clinical evaluation

There were no significant differences in age between HDP and controls [*F*(1, 34) = 0.030, *p* = 0.86], or HDR and controls [*F*(1, 35) = 0.005, *p* = 0.94]. Neither did the education level differ between HDP and controls [*F*(1, 34) = 0.15, *p* = 0.69] or HDR and controls [*F*(1, 35) = 0.008, *p* = 0.94]. No differences in gender were observed in comparing HDP and controls [χ^2^(1) = 0.00, *p* = 1.00] or HDR and controls [χ^2^(1) = 0.012, *p* = 0.90]. The control groups’ intellectual levels were similar to those of HDP [*F*(1, 34) = 0.004, *p* = 0.94] and HDR [*F*(1, 35) = 1.80, *p* = 0.18]. Descriptive data are provided in Table 1.

### Psychopathological and neuropsychological assessment

Data regarding the neuropsychological, cognitive, and motor performance of the participants are displayed in Table 2.

**Table 2:** **Psychopathological and neuropsychological data**.

	HDP (*n* = 18)	HDP–CTR (*n* = 18)	HDP vs. CTR	HDR (*n* = 19)	HDR–CTR (*n* = 18)	HDR vs. CTR
Intellectual level	89.1 (8.4)	90.2 (11.4)	NS	89.5 (11.1)	92.9 (8.6)	NS
HDFCS	11.8 (1.5)					
BDI-II	11.7 (8.4)	4.3 (2.0)	0.001	4.05 (3.0)	4.2 (1.6)	NS
HAM-A	7.2 (3.3)	1.8 (1.2)	0.00	3.3 (1.6)	1.5 (1.2)	0.003
MOCA total score	24.9 (2.6)	27.7 (1.4)	0.003	27.7 (1.4)	29.2 (1.2)	0.001
IFS total score	19.6 (4.6)	23.6 (1.5)	0.001	23.9 (1.9)	26.0 (2.0)	0.003
Motor series	2.5 (0.7)	2.8 (0.3)	NS	2.8 (0.3)	3.0 (0.0)	NS
Conflicting instructions	2.1 (1.1)	2.8 (0.3)	0.01	2.8 (0.3)	2.9 (0.2)	NS
Go–no go	1.7 (1.0)	2.6 (0.6)	0.006	2.6 (0.5)	2.7 (0.4)	NS
Backward digits span	2.9 (0.8)	3.1 (0.7)	NS	3.2 (0.8)	4.0 (1.1)	0.01
Verbal working memory	1.7 (0.5)	2.0 (0.0)	0.04	2.0 (0.0)	1.9 (0.2)	NS
Spatial working memory	1.3 (1.3)	2.0 (0.5)	NS	2.1 (0.6)	3.1 (0.8)	0.003
Abstraction capacity	2.1 (0.9)	2.6 (0.4)	0.03	2.6 (0.4)	2.6 (0.4)	NS
Verbal inhibitory control	5.1 (1.3)	5.5 (0.7)	NS	5.5 (0.7)	5.4 (0.9)	NS
Stroop test (W)	65.3 (24.5)	84.0 (15.3)	0.01	84.3 (14.9)	91.8 (7.9)	NS
Stroop test (C)	48.0 (23.5)	63.0 (10.4)	0.01	62.6 (10.2)	62.9 (12.5)	NS
Stroop test (W/C)	24.6 (17.8)	31.7 (12.1)	NS	31.5 (11.8)	38.7 (14.2)	NS
Similarities subtest	18.0 (2.7)	18.2 (4.4)	NS	18.1 (4.3)	19.3 (3.2)	NS

*It represents the overall results for all groups*.

*HDP, Huntington’s disease patients; HDR, relatives; UHDRS, Unified Huntington’s Disease Rating Scale; HDFCS, Total Functional Capacity Scale; BDI-II, Beck Depression Inventory-II; HAM-A, Hamilton Anxiety Rating Scale; IFS, INECO frontal screening; W, word; C, color, W/C, word/color*.

#### HD patients

Relative to controls, HDP evidenced higher levels of anxiety [*F*(1, 34) = 40.19, *p* < 0.01], as measured by the HAM-A. In addition, HDP showed higher levels of depression symptoms [*F*(1, 34) = 12.73, *p* < 0.01].

Also, as compared to their controls, HDP had lower total scores on the MOCA [*F*(1, 34) = 15.94, *p* < 0.01] and the IFS [*F*(1, 34) = 12.05, *p* < 0.01]. A detailed comparison of performance on the eight IFS subtests indicated that both HDP and HDR exhibited deficits in verbal working memory. HDP also showed impairments in conflictive instructions, motor inhibitory control, and abstraction capacity. In addition, HDP were outperformed by controls in the word [*F*(1, 34) = 7.42, *p* < 0.05] and color naming [*F*(1, 34) = 6.09, *p* < 0.05] conditions of the Stroop test. However, no differences were observed in the word/color condition [*F*(1, 34) = 1.9, *p* = 0.16] or the similarities subtest [*F*(1, 34) = 0.01, *p* = 0.89]. See Table 2 for further details.

#### Relatives

Higher levels of anxiety were also observed in HDR [*F*(1, 35) = 15.92, *p* < 0.01] as compared to their controls. However, no differences between HDR and controls were observed in the BDI-II total score [*F*(1, 35) = 0.84, *p* < 0.77].

Huntington’s disease patients’ relatives had lower total scores than controls in both the MOCA [*F*(1, 35) = 11.36, *p* < 0.01] and the IFS [*F*(1, 35) = 9.67, *p* < 0.01]. Analysis of the eight IFS subtests revealed verbal working memory impairments in HDR. No differences between groups were observed in the word [*F*(1, 35) = 3.61, *p* = 0.06], color [*F*(1, 35) = 0.06, *p* = 0.93], or word/color [*F*(1, 35) = 2.80, *p* = 0.10] conditions of the Stroop test. Both groups also obtained comparable scores on the similarities subtest [*F*(1, 35) = 1.02, *p* = 0.31]. See Table 2 for further details.

### Action–language in HDP

#### ACE is impaired in HDP

**Figure 1:**
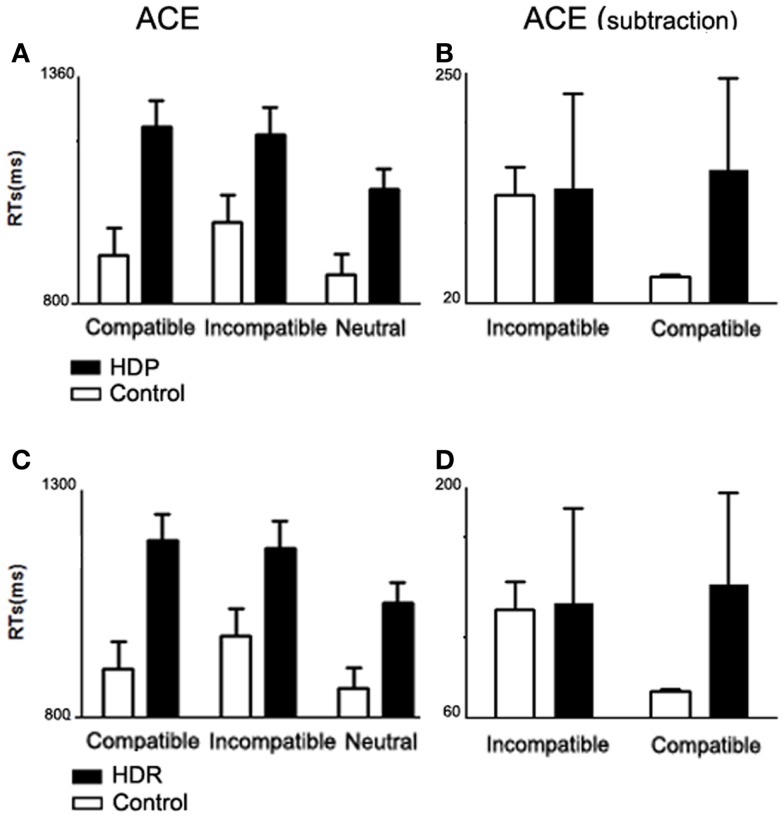
**Action compatibility effect in HDP and HDR**. **(A)** Mean RTs from compatible, incompatible, and neutral trials for HDP. HDP did not show an ACE (compatible facilitation and incompatible delay of RTs). **(B)** ACE subtraction, group comparison of ACE normalized by subtracting mean RT from the neutral trials from the mean RTs from the compatible and incompatible trials. **(C)** Mean RTs for HDR participants. HDR did not show ACE. **(D)** ACE subtraction. In all panels, the bars depict the SD.

We observed (Figure 1A) an interaction of Group × Compatibility [*F*(1, 34) = 8.38. *p* < 0.01]. *Post hoc* comparisons (Tukey’s HSD test, MS = 3560.4; DF = 68.00; η_p_^2^ = 0. 2) revealed an ACE in controls: incompatible trials elicited longer RTs than both compatible (*p* < 0.002) and neutral trials (*p* < 0.0002). Conversely, ACE was abolished in HDP: RT differences were observed only between the neutral trials and the compatible (*p* < 0.0002) and incompatible trials (*p* < 0.0002), there being no differences between compatible and incompatible trials (*p* = 0.93). These results suggest that motor impairment affects action–language processing (Table 3).

**Table 3:** **ACE-RTs**.

Condition group	Compatible (mean ± SD, ms)	Incompatible (mean ± SD, ms)	Neutral (mean ± SD, ms)
HDP	1235.67 ± 380.25	1216.87 ± 383.67	1082.32 ± 289.42
Control	920.01 ± 92.67	1001.35 ± 118.31	872.82 ± 91.29

*Mean and SD of each condition in HDP*.

##### Subtraction analysis

In order to assess performance while controlling for general differences between HDP and controls, neutral RTs were subtracted from compatible and incompatible categories. After subtraction (Figure 1B), group differences became larger [Group × Compatibility interaction, *F*(1, 34) = 11.699. *p* < 0.002]. In particular, the control group showed a larger difference between compatible (*M* = 47.32 ms, SD = 1.38) and incompatible trials (*M* = 128.53 ms, SD = 27, *p* < 0.0002), whereas in HDP the means for compatible (*M* = 153.35 ms, SD = 90.83) and incompatible (*M* = 134.55 ms, SD = 94.27) trials were quite similar (*p* = 0.6).

#### Preserved ACE motor responses to linguistic variables in HDP

##### Stimulus content analysis

Importantly, both HDP and controls responded faster to N sentences than to OH and CH sentences [*F*(2, 68) = 19.919; *p* < 0.00001]. *Post hoc* comparisons (Tukey’s HSD test, MS = 871.44, DF = 68.00) yielded significant differences between N and OH (*p* < 0.002) and between N and CH (*p* < 0.002). No difference was detected between hand-shape sentences (OH vs. CH; *p* < 0.97) (Table 4). This result confirms that motor impairment in HDP was not so severe as to preclude effects of linguistic variables. Consequently, the ACE deficits in HD cannot be explained by a general motor or language impairment.

**Table 4:** **Mean and SD of each sentence list in HDP**.

Sentences group	OHS (mean ± SD, ms)	CHS (mean ± SD, ms)	NS (mean ± SD, ms)
HDP	1215.7 ± 379.4	1213.5 ± 387.6	1167.5 ± 370.98
Control	945.02 ± 97.1	950.02 ± 99.1	918.6 ± 96.35

### Action–language in HDR

#### ACE is impaired in HDR

We identified (Figure 1C) an interaction of Group × Compatibility between HDR and control groups [*F*(2, 70) = 6.32, *p* < 0.01]. *Post hoc* comparisons (MS = 1843.0; DF = 70.00) showed that HDR had shorter RTs on neutral trials as compared to compatible (*p* < 0.002) and incompatible trials (*p* < 0.002); however, they showed no differences between compatible and incompatible trials (*p* < 0.99) (Table 5). These results show that there is no ACE in HDR, suggesting an impairment in the use of the motor system to simulate language content.

**Table 5:** **ACE-RTs**.

Condition group	Compatible (mean ± SD, ms)	Incompatible (mean ± SD, ms)	Neutral (mean ± SD, ms)
Relatives	995.57 ± 265.15	1003.6 ± 276.82	901.4 ± 248.9
Control	950.46 ± 168.27	1027.67 ± 196.42	877.03 ± 157.23

*Mean and SD of HDR conditions*.

#### Subtraction analysis

In order to examine the performance of HDR and their controls while controlling for general group differences, neutral RTs were subtracted from compatible and incompatible categories. After subtraction (Figure 1D), group differences became larger [Group × Compatibility interaction, *F*(1, 35) = 23.669; *p* < 0.00002]. In particular, the control group showed a larger difference between compatible (*M* = 73.42 ms, SD = 70.83) and incompatible trials (*M* = 150.66 ms, SD = 90.18, *p* < 0.0002), whereas in HDR the means for compatible (*M* = 94.16 ms, SD = 51.09) and incompatible (*M* = 102.20 ms, SD = 53.05) trials were similar (*p* = 0.5).

#### Preserved ACE motor responses to linguistic variables in HDR

Both HDR and their controls responded faster to N sentences than to OH and CH sentences [*F*(2, 70) = 22.611, *p* < 0.00001]. *Post hoc* comparisons (Tukey’s HSD test, MS = 50,068, DF = 35.431) yielded significant differences between N and CH (*p* < 0.01). No differences were observed between hand-shape sentences (OH vs. CH, *p* < 0.50; N vs. OH, *p* < 0.23) (Table 6). As in HDP, this effect confirms that motor impairment in HDR was not so severe as to preclude effects of linguistic variables. Consequently, as was the case in HDP, the ACE deficits in HDR cannot be explained by a general motor or language impairment.

**Table 6:** **Mean and SD of each sentence list in HDR**.

Condition group	OHS (mean ± SD, ms)	CHS (mean ± SD, ms)	NS (mean ± SD, ms)
HDR	971.7 ± 267.8	983.9 ± 267.8	956.2 ± 255.6
Control	962.9 ± 170.3	974.25 ± 175.4	935.8 ± 168.4

### Verbal (KDT) and noun (PPT) processing

**Figure 2:**
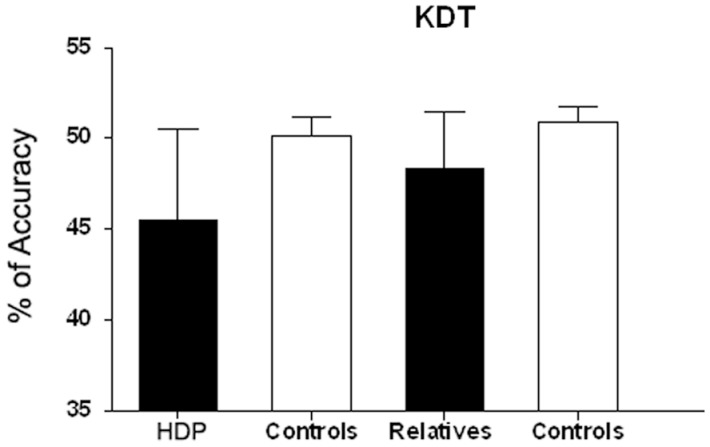
**Verbal processing (KDT) in HDP and RHD**. KDT scale denotes percentage of correct responses (percentage accuracy) in HDP vs. controls. **p < 0.0005; HDR vs. controls, *p* < 0.002; HDP vs. HDR,*p < 0.01.

Having found significant group differences in the KDT [*F*(3, 69) = 11.270; *p* < 0.00001], we performed ANOVAs for each group comparison: HDP vs. HDCTR [*F*(1, 34) = 14.842; *p* < 0.0005], HDR vs. HDRCTR [*F*(1, 35) = 11.400; *p* < 0.001], and HDP vs. HDR [*F*(1, 35) = 4.2285, *p* < 0.05]. The KDT score (percentage of correct responses) was significantly reduced in HDPs (*M* = 45.50; SD = 0.85) as compared to their controls (*M* = 50.16; SD = 0.85). The same was true of HDR (*M* = 48.31; SD = 0.53) relative to their own controls (*M* = 50.88, SD = 0.54). This result suggests specific action–language impairment in both the HDP and HDR groups (Figure 2).

An ANOVA on the PPT scores revealed differences between HDP and their controls [*F*(1, 34) = 6.526; *p* < 0.01], but not between HDR and their controls (*p* = 0.5).

### Correlations

To evaluate whether executive function performance would explain the observed action–language profiles, we performed Spearman’s rank correlations between the ACE and IFS total scores. Considering all groups, significant correlation coefficients were obtained between the ACE and IFS total scores (*N* = 72, *R* = 0.3; *p* < 0.05). Nevertheless, no correlation was observed for HDP (*N* = 18, *R* = 0.2; *p* = 0.5) or HDR (*N* = 19, *R* = 0.3; *p* = 0.2). Finally, in HDP, we did not find significant correlation coefficients between ACE and age/years of illness (*R* = 0.12, *p* = 0.3; *R* = 0.05, *p* = 0.9, respectively). In brief, these correlations suggest that executive functions do not explain the HDP and HDR results. In addition, ACE impairments in HDP were most likely unrelated to age/years of illness, although more complex design is needed to further investigate this issue.

## Discussion

This study sought to further current understanding of motor–language coupling in HD families and to establish whether basal ganglia networks are engaged in motor–language coupling. A strong association between action–language comprehension and motor processes has been reported in previous studies assessing normal participants or other motor conditions (Pulvermuller and Shtyrov, 2003; Hauk et al., 2004; Buccino et al., 2005; Tettamanti et al., 2005; Zwaan and Taylor, 2006). However, there is a dearth of studies exploring this issue in HD populations. This study provides novel results relevant for both clinical research on HD and the cognitive neuroscience of action–language.

### Motor–language impairments as an early marker of HD

Motor–language coupling was evaluated by the ACE paradigm at an early-stage of HD. The present ACE results showed shorter RTs for healthy volunteers in the compatible condition, leading to faster, more accurate movement. Action was effectively facilitated by compatible sentences, as previously reported (Fischler and Bloom, 1980; Glenberg and Kaschak, 2002; Borghi et al., 2004; Kaschak et al., 2005; Tseng, 2005; Borreggine and Kaschak, 2006; Zwaan and Taylor, 2006; Havas et al., 2007; Glenberg et al., 2008b; Aravena et al., 2010; Bergen and Wheeler, 2010; de Vega et al., 2013). Conversely, we did not find an ACE in HD subjects (i.e., no facilitation was observed). These disturbances were not associated with the absence of language discrimination or the existence of motor–response variability. Then, the stimulus content analysis showed that, although HDP were slower (but not significantly so), they preserved the ability to discriminate linguistic aspects of the sentences.

For several years, the neural basis of word–meaning processing has been a topic of interest in cognitive neuroscience. Although there is substantial evidence for the involvement of sensory–motor systems in conceptual processing, it is still unclear whether these play a causal role in such a function (Fernandino et al., 2013). Our findings suggest that action–verb processing partially depends on basal ganglia activation, since the ACE was completely abolished in patients with atrophy of those structures (HDP). This result is consistent with previous studies showing reduced ACE in the early-stages of Parkinson’s disease (Amoruso et al., 2013; Cardona et al., 2013; Ibanez et al., 2013), and action–verb impairments in degenerative brain diseases compromising the motor system, such as motor neuron disease (Bak et al., 2001; Cotelli et al., 2006, 2007; Boulenger et al., 2008) and the frontal variant of frontotemporal dementia (Cotelli et al., 2006).

The earliest and most prominent neuropathological changes in HD are found in the neostriatum (Vonsattel et al., 1985). Loss of basal ganglia volume has been reported in pre-clinical cases (Aylward et al., 1994; Harris et al., 1999). Neuroimaging studies have consistently revealed cortico-basal ganglia compromise in pre-clinical HD (Antonini et al., 1996; Paulsen et al., 2004; Rosas et al., 2008) and a decrease in dopamine receptor binding (Antonini et al., 1996; Lawrence et al., 1998b).

Interestingly, we also found that HDP have an impairment in action–verb (KDT) and noun (PPT) processing. Nevertheless, as compared to controls, relatives presented only KDT deficits with preserved PPT performance. The discrepancy in the performance between KDT and PPT tasks may be explained by the pattern of atrophy in patients as compared to their relatives. Anatomically, tasks involving semantic association of nouns (PPT) result in focal activation of the anterior inferior temporal lobe, the parahippocampal gyrus, and the inferior occipital cortex (Vandenberghe et al., 1996; Ricci et al., 1999; Butler et al., 2009). In contrast, semantic processing of verbs (KDT) is linked to basal ganglia, left frontal cortex (Perani et al., 1999; Cappa et al., 2002), and Brodmann’s areas 44 and 45 (Bak et al., 2001). In view of these findings, the detection of early cognitive changes may be better served by a measure of cognitive impairment subsequent to basal ganglia lesions than by instruments tapping functions subserved by frontal cortical areas.

Altogether, the present results provide evidence for the involvement of a motor-related basal ganglia–cortical circuit in the processing of action–language. Given that our HDP were in an early-stage of the disease and that their relatives had no motor symptoms, the observed action–word processing impairment seems prior to motor symptoms.

### Executive functions and ACE

Classical cognitive theories propose that the role of subcortical structures in language processing is limited to executive functions (Sambin et al., 2012). Typically, language disturbances in HDP have been assumed to result from damage to frontostriatal and frontotemporal areas (Nadeau, 2008; Lepron et al., 2009). HDP perform poorly on some neuropsychological tests which are sensitive to frontostriatal dysfunction. In this sense, it should be noted that HDP showed mild cognitive deficits, especially in subtests of the IFS – namely, conflictive instructions, Go–No Go, verbal and spatial working memory, and abstraction capacity. Nevertheless, these deficits seem not to be directly related to ACE, since there were no correlations between ACE results and total IFS scores in either HDP or HDR. Thus, our results suggest that ACE impairments in HDP and their relatives were related to a *sui generis* motor–language coupling, independent of executive function involvement. Further research is required to clarify to what extent motor–language processing is unrelated to other deficits in HD families.

### Motor–language impairments as a marker of HD vulnerability

Nowadays, genetic tests can establish the presence of the mutation causing HD. However, the diagnosis of HD is still based on clinical evidence, such as outward signs and family history. As HD is genetically transmitted, the children of an affected individual have 50% chance of inheriting the abnormal huntingtin gene and eventually developing the disease (Folstein et al., 1985).

Vulnerability to HD means that individuals with a family history of HD have a high probability of developing the disease or some unspecific deficits related to it (Panegyres and Goh, 2011). The CAG repeat length normally varies from 6 to 35 CAG units. Repeat lengths from 27 to 35 are considered “high normal” and may expand in subsequent generations (Rubinsztein et al., 1996; Quarrell et al., 2007). Repeat lengths from 36 to 39 exhibit reduced penetrance, with manifestations occurring at a later age or not at all (ACMG/ASHG statement) (McNeil et al., 1997; The American College of Medical Genetics/American Society of Human Genetics Huntington Disease Genetic Testing Working Group, 1998; Quarrell et al., 2007). Alleles with 40 repeats are fully penetrant and inevitably associated with progressive motor, cognitive, and behavioral features of HD (Hendricks et al., 2009). It has been observed that longer CAG repeat expansions are associated with earlier disease manifestation (Duyao et al., 1993; Stine et al., 1993; Langbehn et al., 2010), and that age of onset varies considerably for any given CAG repeat expansion (Dennhardt and LeDoux, 2010). Nevertheless, there is growing evidence that cognitive changes occur in individuals who carry an expanded allele prior to the clinical (motor) diagnosis of HD (Dorsey, 2012). Also, recent studies suggest that environmental factors can modify the onset and progression of HD (van Dellen et al., 2005). Moreover, clinical and neuropsychiatric manifestations have been reported in relatives of HDR, irrespective of whether they were HD gene carriers or not (Markianos et al., 2008).

In this study, we showed that HDP and their first-degree relatives all performed poorly on the ACE task. Interestingly, the ACE task results for HDR fall right between those of HDP and controls. The HDR group represents familial HD subjects at risk of developing HD, who did not receive genetic evaluation. Hence, this group would include both HD gene carriers and non-carriers. In this context, an intermediate pattern in cognitive differences between subjects with HD gene expression and subjects at risk without the HD gene could mean that part of the subjects will not develop HD in the future. Nevertheless, familial vulnerability has been reported even in the absence of HD genetic alleles (Markianos et al., 2008; Dorsey, 2012), and previous studies failed to find differences in cognition between prodromal carriers and mutation negative relatives in HD (Blackmore et al., 1995; Giordani et al., 1995; Campodonico et al., 1996, 1998; de Boo et al., 1999; Soliveri et al., 2002). The asymptomatic relatives assessed here represent a group with vulnerability to HD or some unspecific-related deficits (Panegyres and Goh, 2011). Although this group might include both HD gene carriers and non-carriers, its performance in action–language was significantly lower than controls, which suggests that even non-carriers may have selective impairments. Our data are consistent with previous studies on HD reporting other cognitive deficits without clinical motor signs (Henley et al., 2008; Tabrizi et al., 2009), and with findings of familial vulnerability factors even in the absence of HD mutation (Markianos et al., 2008; Dorsey, 2012). Although the probability of being a non-manifest carrier is 50%, all participants in this group were subclinical; however, even non-carriers can present vulnerability factors. Thus, two levels of vulnerability (one represented by gene-carrier relatives with subclinical manifestations, and another by non-carrier relatives with diffuse vulnerability factors) might be reasonably proposed. Taken together, these data indicate that action–language, in general, and the ACE paradigm, in particular, might tap familial vulnerability to HD.

### Relevance for theoretical models of subcortical involvement in motor–language coupling

Classically, language production and comprehension have been related to brain networks in the left inferior frontal and superior temporal cortices, such as Wernicke’s and Broca’s areas (Blank et al., 2002). However, a growing body of clinical and neuroimaging evidence shows that language processing activates a much more complex and widely distributed network (Mesulam, 1990; Price et al., 1996; Pulvermuller, 1999, 2002, 2005).

It is well-established that the motor system plays a fundamental role in action–verb comprehension/production (Pulvermuller, 2005; Pulvermuller and Fadiga, 2010). There is abundant evidence showing that the processing of action–verbs implicates the frontal and motor cortices (Federmeier et al., 2000; Pulvermuller et al., 2001; Yokoyama et al., 2006; Boulenger et al., 2008; Cappelletti et al., 2008; Kemmerer et al., 2008; Tomasino et al., 2008). However, numerous studies have failed to find a somatotopic distribution of action–verbs or sentences in the motor cortex (Aziz-Zadeh et al., 2006; Ruschemeyer et al., 2007; Postle et al., 2008; Raposo et al., 2009). Accordingly, theories of embodied cognition propose that language comprehension is based on perceptual and motor processes (Bak, 2013). For its own part, a weak view of the embodied cognition hypothesis proposes that other cortical regions are indeed required (Brass et al., 2007). Besides, evidence from Parkinson’s disease studies suggests an intricate connection between language and the motor system by a bidirectional influence of motor and language areas, including subcortical motor areas and even non-motor regions (Bak, 2013; Cardona et al., 2013).

The primary spot on HD neuropathology is the basal ganglia. Hence, this disorder provides an important model for the role of the human basal ganglia in motor–language coupling. Atrophy of caudate and putamen nuclei in HDP is a well-established fact (Vonsattel et al., 1985; Mann et al., 1993; Loh et al., 1994; Aylward et al., 1997). This degenerative process is present even in the early-stages of the disease (Antonini et al., 1996), and it has been reported in some studies in pre-symptomatic individuals who carry the HD mutation (Aylward et al., 1994). Some frontal neocortical atrophy may also occur later in the course of the disease (Aylward et al., 1998). Therefore, early HDP and HDR constitute ideal models to study the role of subcortical structures in motor–language coupling.

Selective action–verb impairments (using non-motor linguistic tasks, such as action naming) in other motor diseases have been reported in progressive supranuclear palsy, amyotrophic lateral sclerosis, and cortico-basal degeneration. Instead, the ACE paradigm may offer more sensitive, discriminatory measurements of action–language interaction already reported in Parkinson’s disease (Ibanez et al., 2013) and now in Huntington families. Recently, Cardona et al. (2014) showed that ACEs are abolished in Parkinson’s disease, but not in neuromyelitis optica and acute transverse myelitis (two models of preserved brain motor areas and musculoskeletal system injury). Additional comparative studies including other brain-affected (e.g., progressive supranuclear palsy, amyotrophic lateral sclerosis) and musculoskeletal-affected motor diseases (e.g., glutamine expansion diseases such as muscle–spinal atrophy) would expose which disorders present action–language impairments as reported with the ACE task.

Available evidence indicates that the basal ganglia participate in multiple parallel segregated circuits or “thalamo-cortical loops” involving connections with motor, sensory, and cognitive areas of the cerebral cortex (Alexander et al., 1986; Hoover and Strick, 1993; Middleton and Strick, 2002). The motor network is a complex circuit that includes primary motor and sensory cortices, pre-motor, parietal, precuneal, and dorsal lateral pre-frontal cortical regions, the basal ganglia, and the cerebellum (Cardona et al., 2013). The present findings support the notion that motor system involvement during language processing engages subcortical areas. Action–language seems to rely not only on the motor cortex, but also on neuronal circuits involving the basal ganglia network.

### Limitations and directions for further research

One limitation of this work is that the HDR group did not receive genetic screening. Thus, it may have included both genetic pre-symptomatic and healthy relatives without HD genetic heredity. Nevertheless, familial vulnerability has been reported even in the absence of HD genetic alleles (Markianos et al., 2008; Dorsey, 2012). Therefore, the HDRs in this study represent a vulnerability group including pre-symptomatic HDP as well as individuals with a diffuse vulnerability, not restricted to HD expanded alleles. Further studies are needed to explore differences in action–language impairments between relatives with and without HD non-expanded alleles.

Also, it would be useful to elaborate on this study by quantifying the ACE task according to the number of triplets’ expansion. Longitudinal studies that evaluate disease development based on ACE results could support the role of the ACE task as a predictor of HD onset.

## Conclusion

The ACE task unmasked the initiation of action–language deficits subsequent to basal ganglia network damage. To our knowledge, this is the first study showing that motor–language coupling is impaired in HD relatives. These findings highlight the key role of a cortico-basal ganglia network in motor–language impairment – a distinct cognitive deficit in HD.

This overall result has important clinical implications. There is increasing evidence that cognitive impairments precede the phenotypic expression of HD. Studies describing the transition from health to disease phenotype are important to understand the nature of the disease and to outline possible therapies for different stages of the disease.

Here, we established that the ACE task could be useful to uncover asymptomatic cognitive dysfunction in HD, since the ACE is impaired in HDR preceding other cognitive and motor impairments. Our findings demonstrate that the ACE paradigm constitutes a sensitive method for the assessment of subcortical cognitive damage, which may be of critical importance for neurocognitive biomarker research, as well as for drug testing in clinical trials.

## Conflict of Interest Statement

The authors declare that the research was conducted in the absence of any commercial or financial relationships that could be construed as a potential conflict of interest.
